# Integrated EIT system for functional lung ventilation imaging

**DOI:** 10.1186/s12938-019-0701-y

**Published:** 2019-07-25

**Authors:** Geuk Young Jang, Ghazal Ayoub, Young Eun Kim, Tong In Oh, Chi Ryang Chung, Gee Young Suh, Eung Je Woo

**Affiliations:** 1 Department of Biomedical Engineering, Graduate School, Kyung Hee University, Yongin, South Korea; 2 Department of Biomedical Engineering, College of Medicine, Kyung Hee University, Seoul, South Korea; 3 BiLab, Seoul, South Korea; 4 Department of Critical Care Medicine, Samsung Medical Center, Sungkyunkwan University School of Medicine, Seoul, South Korea

**Keywords:** Electrical impedance tomography, Functional lung ventilation imaging, Mechanical ventilation, Integrated approach, Real-time bedside imaging

## Abstract

**Background:**

Electrical impedance tomography (EIT) has been used for functional lung imaging of regional air distributions during mechanical ventilation in intensive care units (ICU). From numerous clinical and animal studies focusing on specific lung functions, a consensus about how to use the EIT technique has been formed lately. We present an integrated EIT system implementing the functions proposed in the consensus. The integrated EIT system could improve the usefulness when monitoring of mechanical ventilation for lung protection so that it could facilitate the clinical acceptance of this new technique.

**Methods:**

Using a custom-designed 16-channel EIT system with 50 frames/s temporal resolution, the integrated EIT system software was developed to implement five functional images and six EIT measures that can be observed in real-time screen view and analysis screen view mode, respectively. We evaluated the performance of the integrated EIT system with ten mechanically ventilated porcine subjects in normal and disease models.

**Results:**

Quantitative and simultaneous imaging of tidal volume (TV), end-expiratory lung volume change ($\triangle$EELV), compliance, ventilation delay, and overdistension/collapse images were performed. Clinically useful parameters were successfully extracted including anterior/posterior ventilation ratio (A/P ratio), center of ventilation (${\mathrm{CoV}}_{{x}}$, ${\mathrm{CoV}}_{{y}}$), global inhomogeneity (GI), coefficient of variation (CV), ventilation delay and percentile of overdistension/collapse. The integrated EIT system was demonstrated to suggest an optimal positive end-expiratory pressure (PEEP) for lung protective ventilation in normal and in the disease model of an acute injury. Optimal PEEP for normal and disease model was 2.3 and $7.9 \, {\mathrm{cmH}}_{2}\mathrm{O}$, respectively.

**Conclusions:**

The proposed integrated approach for functional lung ventilation imaging could facilitate clinical acceptance of the bedside EIT imaging method in ICU. Future clinical studies of applying the proposed methods to human subjects are needed to show the clinical significance of the method for lung protective mechanical ventilation and mechanical ventilator weaning in ICU.

## Background

Electrical impedance tomography (EIT) is a noninvasive imaging method to produce cross-sectional images of electrical conductivity distributions inside the human body [1–5]. Among numerous clinical applications, time-difference EIT has been used for real-time bedside imaging and monitoring of a mechanically ventilated patient in intensive care units (ICU) [6–12]. During mechanical ventilation, it is important to recruit collapsed parts without causing overdistension of other parts of the lungs. The so-called lung protective ventilation (LPV) requires continuous bedside imaging of regional lung ventilation in response to a ventilator setting and/or a posture of the patient. Proper interpretation of functional lung ventilation images is essential to search for an optimal patient-specific ventilator setting including respiration rate (RR), airway pressure, tidal volume (TV), positive end-expiratory pressure (PEEP), end-expiratory lung volume (EELV), global and regional lung compliances, posture, and other respiratory mechanics parameters [13, 14].

Considering that EIT could be a potential solution to this clinical problem, numerous attempts have been made to produce and interpret functional EIT images of lung ventilation using custom-designed or commercial EIT devices [15–17]. To find an optimal ventilator setting, various criteria have been proposed and tested in experimental studies of animal and human subjects [18, 19]. Though new clinical findings have been progressively utilized for LPV of patients in ICU and also to treat patients with pulmonary dysfunction [20, 21], there was no standard and/or clear definitions of parameters needed to accelerate clinical acceptance. Following the significant efforts of several concerted actions [19, 22–25], the interdisciplinary group called TRanslational EIT developmeNt stuDy (TREND) was recently formed and built a consensus for clinical applications of lung EIT by comparing and analyzing related studies over the past 30 years [26].

We proposed the integrated EIT approach consisting of the customized EIT system and new visualization platform. It displays five functional images and six EIT measures together which was suggested by the TREND consensus for describing the lung status of a mechanically ventilated patient. We presented at the two operating modes. In the real-time screen view, we can monitor pulmonary function based on the air volume changes over time. The analysis screen mainly views tissue characteristics for overdistension/collapse for finding optimal ventilator settings. We validated the EIT measures and functional EIT images in the integrated method with experimental data obtained from normal and disease animal model.

We will first describe the summary of animal experimental results using an EIT device whose performance was fully validated [27–31] and the integrated platform with 11 functional imaging methods and associated parameters briefly. Then, we will discuss the pros and cons of the integrated EIT system and suggest future clinical study designs.

## Results

### Tidal volume changes in normal porcine lungs

**Fig. 1:**
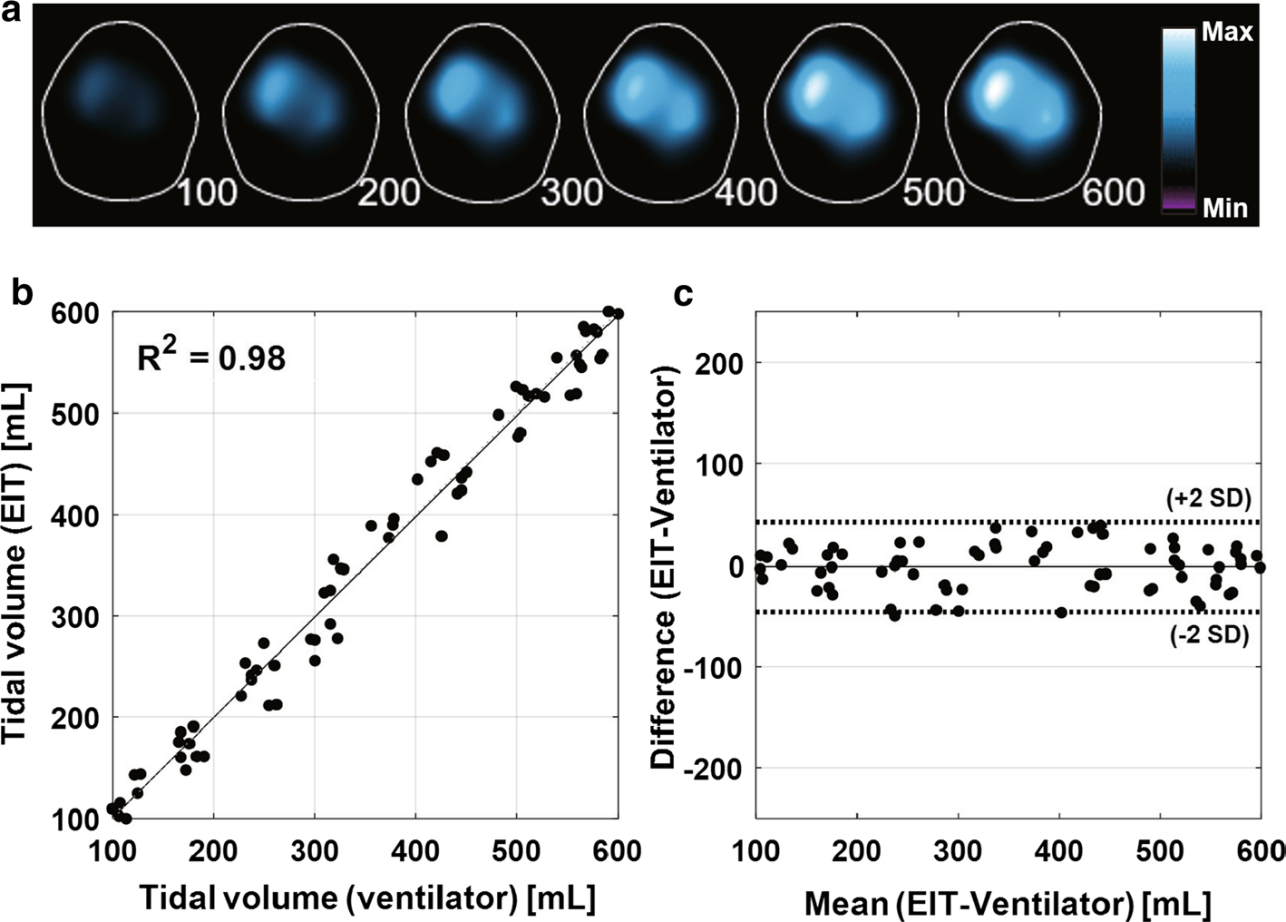
TV images and TV values extracted from the images. **a** TV images of a mechanically ventilated pig with 6 different tidal volume settings. **b** Linear relation between TV from the reconstructed TV images and the air volume supplied from the mechanical ventilator. **c** Bland–Altman plot with ± 2 standard deviation (SD)

During volume-controlled mechanical ventilation for 10 normal pigs, TV was adjusted at 6 different levels from 100 to 600 mL at $0~{\mathrm{cmH}}_2\mathrm{O}$ PEEP. Fig. 1a shows typical TV images calibrated with the applied air volumes from the mechanical ventilator. The correlation analysis in Fig. 1b showed ${\mathrm{R}}^2$ value of 0.98 between TV values from the reconstructed images and the supplied air volumes from the mechanical ventilator. The repeatability was assessed by the Bland–Altman analysis as shown in Fig. 1c with less than 6% mean error between these two measurements. The Bland–Altman plot indicates that the calibrated TV image implemented in the integrated EIT system can be used as a reliable measure of the true tidal volume.

### Ventilation functions from normal porcine lungs during PEEP titration

**Fig. 2:**
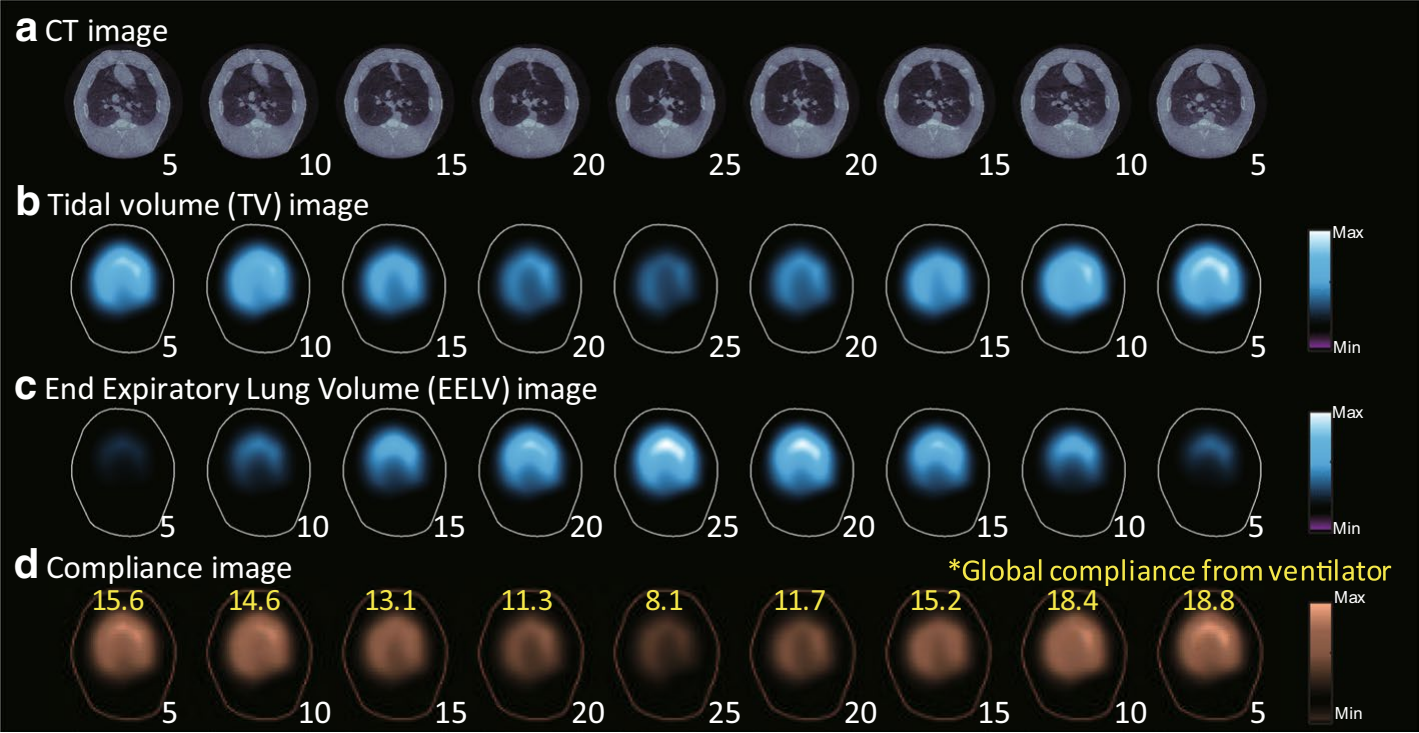
Functional EIT images during PEEP titration in a normal pig. **a** CT images at PEEP levels of 5, 10, 15, 20, 25, 20, 15, 10, and $5~{\mathrm{cmH}}_2\mathrm{O}$. **b**–**d** are functional EIT images of TV, $\triangle$EELV, and compliance, respectively

Different PEEP values were sequentially applied to normal pigs at 0, 5, 10, 15, 20, 25, 20, 15, 10, 5, and $0 ~{\mathrm{cmH}}_2 \mathrm{O}$. The chest X-ray CT images of the pig in Fig. 2a were obtained after setting each PEEP value to confirm its effects. Note that the lungs were progressively more and less inflated as the PEEP value was increased and decreased, respectively. Figure 2b–d shows the reconstructed functional images of TV, $\triangle$EELV, and compliance, respectively, during the PEEP titration. Ventilation delay image was confirmed in the collapse model where the delay difference was clearly shown. As the PEEP value increased, $\triangle$EELV increased as expected. Since increased $\triangle$EELV reduced the lung compliance, the tidal volume decreased at higher values of PEEP. The correlation between the compliance image and the global lung compliance estimated using the pressure/volume data from the mechanical ventilator was high with the ${\mathrm{R}}^{2}$ value of 0.96. These results showed that EIT images could be used to quantitatively measure air distribution in the lungs in real time.

### Ventilation functions from porcine lungs with collapsed parts during PEEP titration

**Fig. 3:**
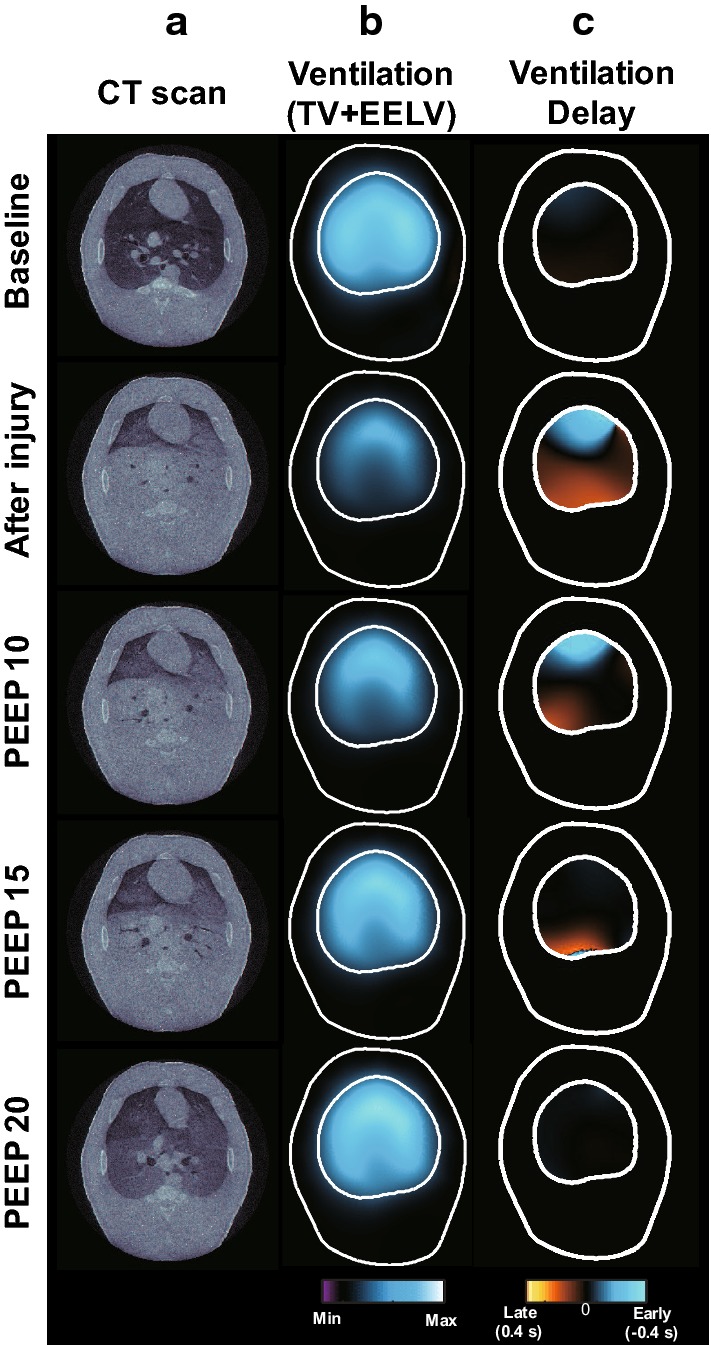
Functional EIT images of the animal model with acute lung injury. **a** X-ray CT, **b** ventilation (TV+EELV), and **c** ventilation delay images are plotted before and after the lung injury at different PEEP levels

The collapse model was used to confirm a significant change in ventilation and ventilation delay. After producing the collapsed part through the saline lavage method, we increased the PEEP from $5 ~ \hbox {to}~ 20~{\mathrm{cmH}}_2\mathrm{O}$. Figure 3 shows the X-ray CT images in (a) and two different EIT functional images in (b) and (c) before and after producing the collapsed parts. When inducing the lung collapse model using saline lavage in the supine posture, the collapse of the alveoli was severely induced in the dorsal region due to gravity.

**Fig. 4:**
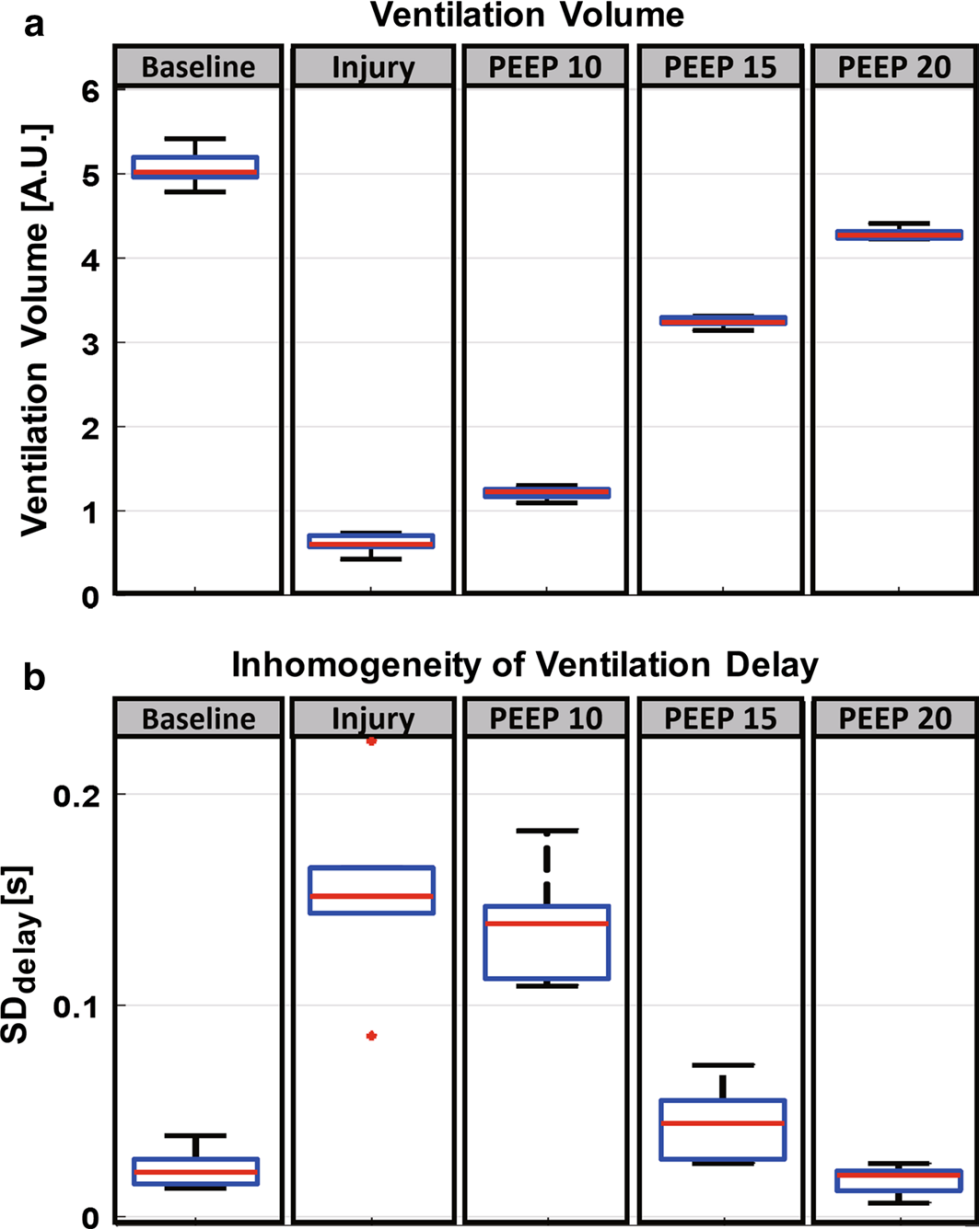
Measures from functional EIT images of the animal model with acute lung injury. **a** Changes in ventilation volume and **b** changes in inhomogeneity of ventilation delay before and after the lung injury in Fig. 3

The X-ray CT images show that the lung collapse occurred in the dorsal region clearly. The EIT ventilation images in (b), expressed as a sum of TV and $\triangle$EELV images, showed decreased pixel values after the injury. As PEEP increased, the collapsed parts were gradually recruited. In the ventilation delay images in Fig. 3c, positive pixel values (blue region) indicate that the corresponding pixels inflated earlier than other pixels. The inhomogeneity of the ventilation delay image after inducing the injury was sharply increased. The air was supplied to the dependent region relatively late compared to the other regions. When the PEEP level was increased, the difference in pixel values within the ventilation delay image was reduced. The degree of homogeneity in regional ventilation delay images has a possibility to be used as an index to evaluate tidal recruitment [32]. From the plots in Fig. 4a, we can see that the lung volume increased rapidly at $15 ~{\mathrm{cmH}}_2 \mathrm{O}$ PEEP and was recovered to 87.1% of the normal value at $20~{\mathrm{cmH}}_2 \mathrm{O}$ PEEP. In Fig. 4b, the inhomogeneity of ventilation delay was increased significantly after inducing the lung injury and decreased as increasing the PEEP level [32].

### Air distributions in porcine lungs with collapsed parts during PEEP titration

**Fig. 5:**
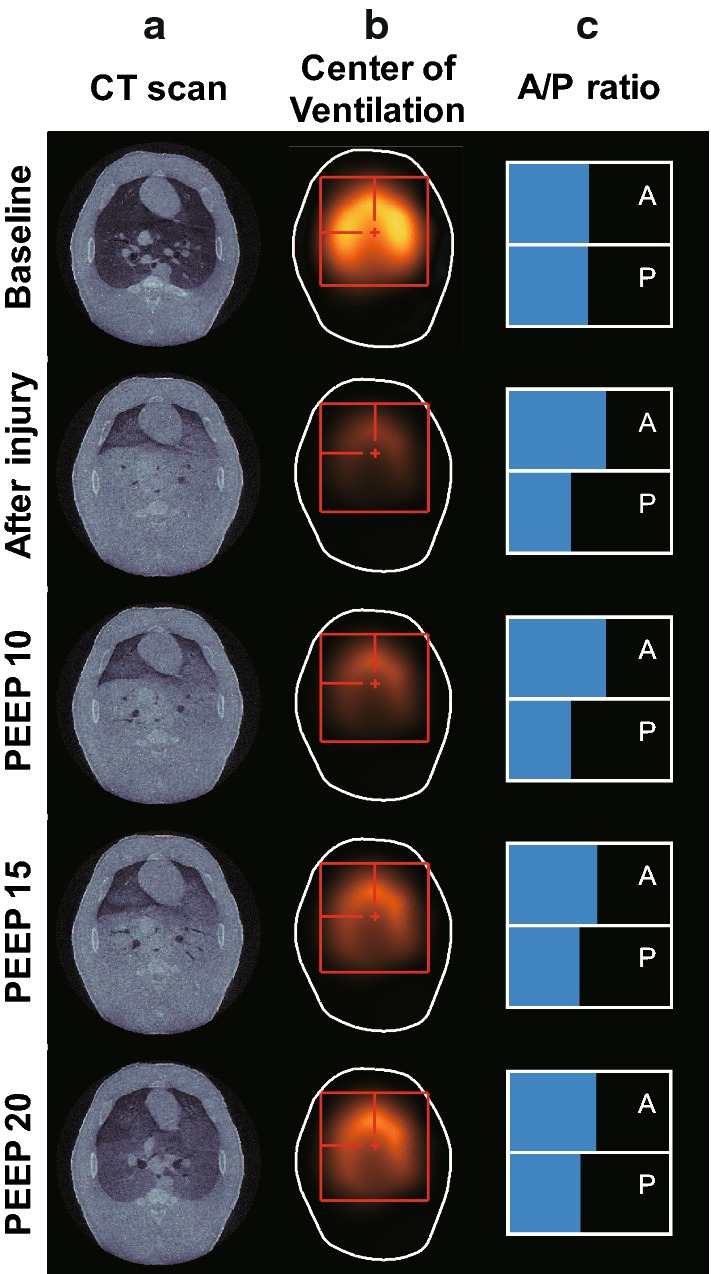
Effects of increased PEEP levels on the air distributions in the porcine lungs with collapsed parts: **a** X-ray CT images, **b** $\mathrm{CoV}$ images, and **c** $\mathrm{A/P}$ ratio

Figure 5 graphically presents the estimated center of ventilation ($\mathrm{CoV}$) and anterior-to-posterior ventilation ratio ($\mathrm{A/P~ratio}$) values of the porcine lungs with collapsed parts during the PEEP titration. Right after inducing the lung injury, the $\mathrm{A/P~ratio}$ was significantly increased by 51.1% because the lung collapse occurred more in the dorsal (posterior) region than in the ventral (anterior) region as confirmed by the X-ray CT images. Similarly, the $\mathrm{CoV}$ was shifted toward the ventral region. As the PEEP level increased, the $\mathrm{A/P~ratio}$ was restored to 91% of the normal value.

**Fig. 6:**
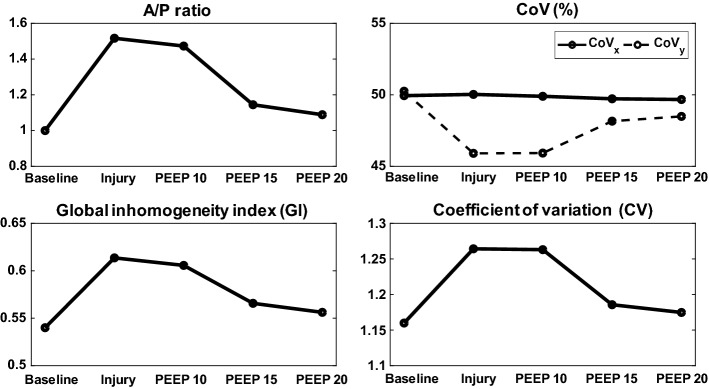
Measures from EIT images of the animal model with acute lung injury. Changes of $\mathrm{A/P}$ ratio, $\mathrm{CoV}$, $\mathrm{GI}$, and $\mathrm{CV}$ of the porcine lungs with collapsed parts during the increasing phase of the PEEP titration

Figure 6 shows how the $\mathrm{A/P}$ ratio and $\mathrm{CoV}$ changed during the PEEP titration. Right after inducing the lung injury, the ${\mathrm{CoV}}_{{y}}$ decreased to 45.3% from its normal value of 50% and then increased back to 48.3% as the PEEP level increased to $20~{\mathrm{cmH}}_2\mathrm{O}$. On the other hand, the ${\mathrm{CoV}}_{{x}}$ changed less than 1%. Both global inhomogeneity index ($\mathrm{GI}$) and coefficient of variation ($\mathrm{CV}$) indices increased immediately after the lung injury and then decreased back to their normal values at $20~{\mathrm{cmH}}_2\mathrm{O}$ PEEP.

### Integrated EIT system

**Fig. 7:**
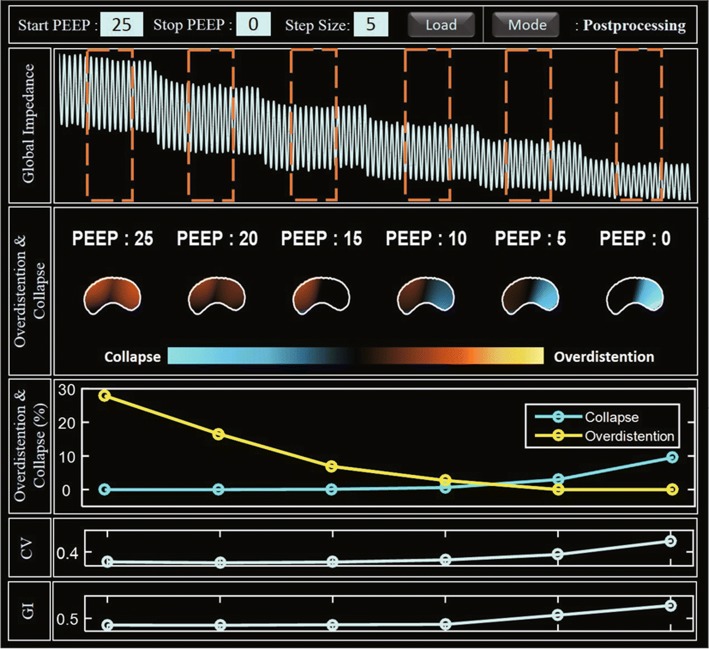
Analysis screen view of the integrated EIT system for the PEEP titration of the lungs with acute injury. Note that the optimal PEEP value is higher than the value of the normal case in Fig. 10 to recruit the collapsed parts due to the injury

The ventilator-induced lung injury (VILI) should be avoided when an increased PEEP level is used to recruit any collapsed parts of the lungs. To select an optimal PEEP level, the regions of collapse and overdistension should be identified and the effects of varying the PEEP level on those regions should be observed. Figure 7 shows the optimal PEEP analysis through the analysis screen view of integrated EIT System in lung collapse model. During the decreasing phase of the PEEP titration from 25 to $0 ~{\mathrm{cmH}}_2\mathrm{O}$ in $5~{\mathrm{cmH}}_2\mathrm{O}$ step, the reconstructed compliance images were analyzed to obtain the maximum compliance at each pixel during the PEEP titration. The airway pressure from the mechanical ventilator at the time of maximum compliance is denoted as ${\mathrm{P}}_{\mathrm{max}}$. At each PEEP value, every pixel is classified as normal, collapse, or overdistension using the compliance difference $\Delta {C}$ between ${C}_{\mathrm{max}}$ and its compliance at the PEEP value. Overdistension and collapse images show how the amounts of the overdistended and collapsed parts of the lungs changed as the PEEP level decreased.

Overdistension and collapse can also be provided as measures according to PEEP titration. The PEEP level of $7.9~ {\mathrm{cmH}}_2\mathrm{O}$ was chosen as the optimal level since it could minimize both overdistension and collapse values. Optimal PEEP selection using air distribution parameters was also provide in the analysis screen view. It shows the change of GI and CV according to PEEP level, and it guides PEEP selection through air distribution inhomogeneity. From these results, GI and CV were significantly reduced from over $10~ {\mathrm{cmH}}_2\mathrm{O}$ PEEP.

**Fig. 8:**
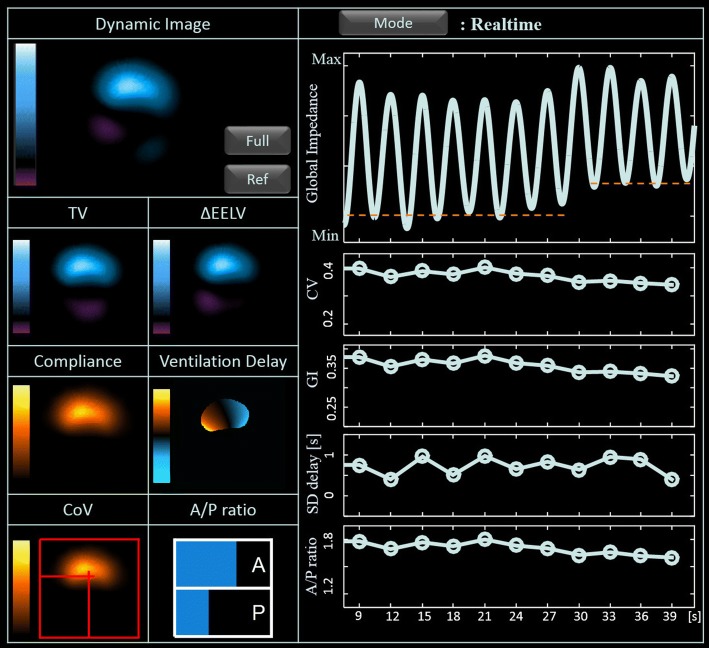
Real-time screen view of the integrated EIT system from the lungs with acute injury

**Fig. 9:**
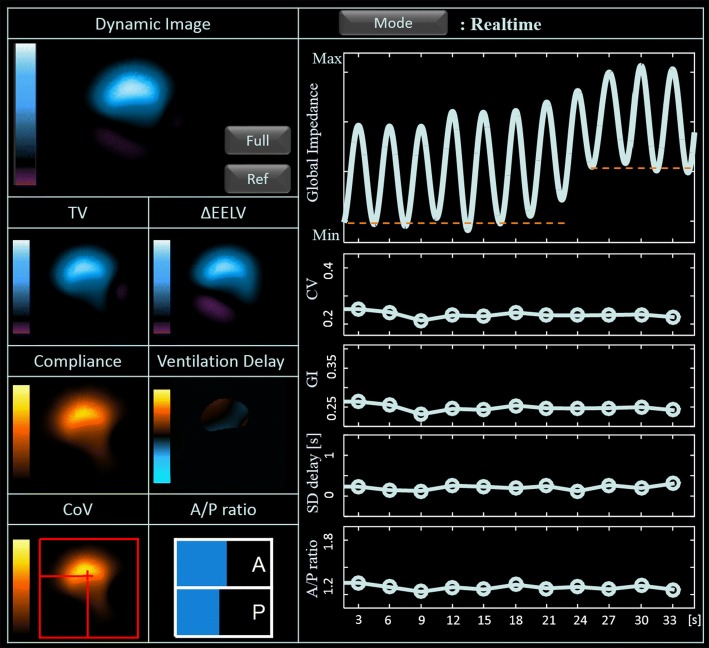
Real-time screen view of the developed integrated EIT system from the normal lungs

After applying the optimal PEEP, the real-time screen view can be used to monitor the recovery of the real-time pulmonary function. Figure 8 shows the results after inducing acute lung injury. The effects of lung injury are shown in the dynamic image and other functional images. Note that the values of CV, GI, SD delay and A/P ratio are increased when compared with those values shown in Fig. 9 before the injury. The optimal PEEP effect can be expected to restore the values to the before injury state.

## Discussion

EIT imaging cannot compete against X-ray CT, for example, in terms of the spatial resolution. The relatively poor spatial resolution of EIT is due to the fundamental limitations of its ill-posed inverse problem. We should, therefore, take full advantage of its high temporal resolution such as 50 frames/s in its clinical applications. Time-difference EIT imaging augmented by integrated functional imaging techniques described in this paper could be a clinically useful bedside image-based monitoring tool especially for lung ventilation imaging during mechanical ventilation.

During mechanical ventilation, the pressure from the mechanical ventilator is adjusted to recruit collapsed parts. Very careful pressure controls are necessary not to produce acute lung injury from overdistending other parts of the lungs. It is, therefore, important to continuously monitor the actual amount of air volume supplied to the lungs and its distributions in the lungs in real time. Though EIT has high potential for providing these image-based monitoring functions, its clinical acceptance is relatively slow. One of the reasons stems from the lack of an integrated approach providing all available information in a quickly readable way. In this paper, we proposed such an integrated approach by implementing eleven functional imaging and measures proposed in the most recent consensus from the interdisciplinary group called TRanslational EIT developmeNt stuDy (TREND) [26].

From the real-time screen view of integrated EIT system, the functional image of TV could be successfully obtained to quantify the air volume supplied to the lungs. The aeration change could be estimated from the $\triangle$EELV obtained at two different PEEP levels. Functional EIT images of compliance and overdistension and collapse clearly change after inducing the lung collapse model. These functional images together with time delay images could clearly localize overdistended and collapsed parts of the lungs. Diagnostic parameters of CoV, A/P ratio, GI, and CV provided useful feedback information during the PEEP titration to recruit collapsed parts of the lungs. This change can also be used for optimal PEEP selection. Within the PEEP titration for finding optimal PEEP, both methods of ventilation homogeneity and tissue characterization can be tried through the analysis screen view of the integrated EIT system. After applying optimal PEEP in clinical trials, validation should be verified by monitoring the change of ventilation characteristic in the real-time screen view. Also, we need to consider the issues of interpreting for the integrated EIT system results. We measured the macroscopic phenomena due to the microscopic changes in the alveoli and airway. Therefore, careful insight is required based on understanding the respiratory mechanics of the lungs and the volume and flow of air inside the lungs [33, 34].

## Conclusion

We proposed the integrated EIT system for functional lung ventilation imaging to enhance its clinical usefulness. The system presented in this paper can be readily used for human subjects in future clinical studies. Applying the proposed integrated approach to different EIT devices, their performance analyses could be conducted. From these future studies, it will be very helpful to produce clinical lung EIT standards in terms of the hardware, software, and clinical practice in bedside real-time image-based monitoring of mechanically ventilated patients. The integrated approach will apply not only lung protective ventilation through optimal patient-specific ventilator setting but also spontaneous breathing monitoring for ventilator weaning. We need to verify the usefulness of monitoring of functional images and EIT measures provided by the integrated approach for ventilator weaning during the spontaneous breathing test [35]. This will further facilitate the clinical acceptance of the technique, which can reduce the risk of mechanical ventilation and also the overall healthcare cost of ICU patients.

## Methods

**Fig. 10:**
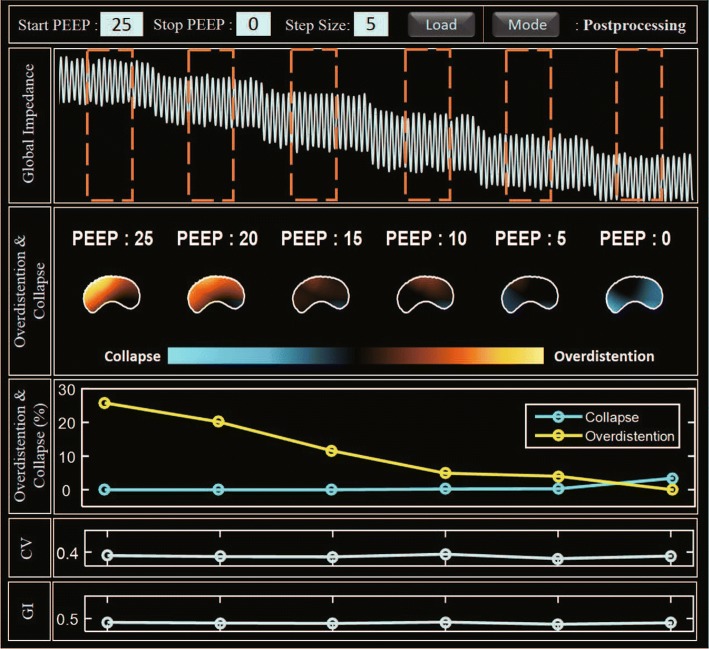
Analysis screen view of the developed integrated EIT system for the PEEP titration of the normal lungs

### Integrated EIT system

Integrated EIT software platform was implemented in PC with the Eclipse environment (JAVA SE 8). The PC was communicated with a 16-channel KHU Mark 2.5 EIT device with adjacent current-injection and voltage-measurement protocol and receiving measured data through USB 2.0 [31]. RS232 serial port was used for storing air volume and pressure data measured from a mechanical ventilator. Functional images and measures were generated through data post-processing as described in the following sections.

The real-time screen view (Fig. 9) shows the pulmonary function change during mechanical ventilation. Dynamic image and global impedance signal were directly calculated from two successive data for the time-series of reconstructed EIT images within 8 ms. The computation time was faster than other processing time for EIT data acquisition and reconstructing tomographic images. These are updated at same speed as the imaging frame rate of the EIT system. In the real-time screen view, four functional images of tidal volume (TV), end-expiratory lung volume change ($\triangle$EELV), compliance, and ventilation delay were computed within 20 ms. These functional images can be updated at every breath. Additionally, we presented $\mathrm{CoV}$ and A/P ratio as figures for easier indication. Four parameters of A/P ratio, GI, CV and ventilation delay were displayed with the format of the time chart. Especially, ventilation delay originally presented in image format, but it was better to display along with time chart as the standard deviation of ventilation delay because it can provide more intuitive interpretation.

When applying PEEP titration which decreases the PEEP level from high to low pressure, we can use the analysis screen view (Fig. 10) for finding optimal mechanical ventilation settings [36]. After decremental PEEP titration, the program shows tissue-classified results (overdistension/collapse) as image and percentile values for each PEEP. Also, CV and GI parameters display together because PEEP titration can be applied with different posture. From the pulmonary function analysis, we could find the optimal PEEP setting.

The EIT data stores the measurement time information and the measured voltage data set in the binary format every 512 frames. Airway volume and pressure data measured from the ventilator are also saved as text format with PC time information.

### TREND consensus

To follow the TREND consensus, time-difference EIT dynamic images are expressed as images of resistivity changes between each time and a chosen reference time. The clinical significance of an EIT image is based on the theoretically and experimentally validated correlation between its pixel value and the air volume at the corresponding location inside the lungs [37, 38]. Different functional images and parameters can be derived from a time-series of EIT images based on this correlation. Table 1 shows the list of functional images and measures adopted in the integrated EIT system among the parameters of the Lung EIT proposed by TREND. These are classified into five categories including three different groups of functional images and two groups of parameters.

**Table 1:** Functional images and measures in lung EIT [26]

Category	Technique	Alternative terms
Functional image		
Ventilation (distribution of)	Tidal volume	*TV*
	Compliance	*C*
	Ventilation delay	VD
Aeration change	Volume-difference	$\triangle$EELV
Tissue classification	Overdistension/collapse	
Parameter		
Characterization of spatial distribution of ventilation	Anterior/posterior ventilation ratio	A/P ratio
	Center of ventilation	${\mathrm{CoV}}_{\mathrm{\mathrm x}}$, ${\mathrm{CoV}}_{{y}}$
	Global inhomogeneity	GI
	Coefficient of variation	CV
Examination-specific measure	Overdistension/collapse	

### Images of ventilation and aeration change

In the real-time screen view, four functional images of lung ventilation are suggested by the TREND consensus including TV, $\triangle$EELV, compliance, and ventilation delay. The TV image is computed as the difference of two EIT images obtained at the end-inspiration and end-expiration times. The end-inspiration and end-expiration times can be detected by searching for peaks and valleys in the time-varying signal derived by summing all pixel values. The sum of all pixel values is called the global impedance signal since it is proportional to the sum of all trans-impedance values across the chest. One TV image can be produced for each breathing cycle and a time-series of TV images expresses ventilation changes over multiple breathing cycles.

The EELV image represents the amount of residual air remaining in the lungs at the end of expiration. The total EELV can be measured using a spirometer with the nitrogen washout or helium dilution techniques. In lung EIT, the aeration image ($\triangle$EELV) is calculated as the difference of two EIT images measured at two end-expiratory times. The $\triangle$EELV images, therefore, represent EELV changes between two end-expiratory times especially during a PEEP titration.

Compliance (${C}$) is the change in volume that occurs per unit change in the pressure [39]. It can be defined as follows for a given lung volume change and pressure change.

1 $$\begin{aligned} {C}=\frac{\Delta {V}}{\Delta {P}} \end{aligned}$$

where $\Delta V$ is the volume change and $\Delta P$ is the pressure change. Assuming that the pressure is homogeneous throughout the entire lungs, the compliance image can be produced by dividing each pixel of the TV image by the pressure difference obtained from the ventilator when acquiring TV image data [40].

Due to resistance and compliance of the regional lung, different regional ventilation delays (RVD) may occur.

2 $${\text{RVD}}\,[\% ]\, = \,\frac{{\Delta t_{j}^{{40\% }} }}{{t_{{\max }} }} - t_{{\min }} \, \times \,100\%,$$

where $\Delta t_{j}^{40\%}$ is the time point at which the impedance of *j* pixel reaches 40% of its maximum value and $t_{\text{max}}-t_{\text{min}}$ is the time difference between start-inspiration ($t_{\text{min}}$) and end-inspiration ($t_{\text{max}}$). The average opening time is calculated in the same way using the global impedance signal that is the sum of all pixel values. The ventilation delay image is formed from the difference image between each pixel for opening time and the average opening time [32]. The unit of ventilation delay was converted from a percentage to the time unit to facilitate comparison with clinical outcomes. In the real-time screen view, we additionally display the standard deviation of ventilation delay calculated from the ventilation delay image as a measure.

### Image for tissue classification

To detect overdistended and atelectatic lung regions, a joint analysis of several EIT images is performed since those regions can be characterized by different values of compliance and TV. To distinguish between these two regions, the following key observation is employed: an increase in pressure decreases the compliance of an overdistended region whereas it increases the compliance of an atelectatic region. While increasing PEEP from 0 to 25 cmH$_2$O in a step of $5~{\mathrm{cmH}}_2 \mathrm{O}$, six compliance images are produced at each PEEP value. This can be also done while decreasing PEEP from 25 to $0~{\mathrm{cmH}}_2 \mathrm{O}$. For each pixel, a plot of six compliance values versus PEEP is obtained, where this pixel compliance curve is concave for most pixels. We set the PEEP value when the maximum compliance ${C}_{\mathrm{max}}$ occurred as ${\mathrm{PEEP}}_{{C}_{\mathrm{max}}}$. While the PEEP value was smaller or larger than ${\mathrm{PEEP}}_{{C}_{\mathrm{max}}}$, the pixel belonged to an atelectatic or overdistended region, respectively. For a pixel at a certain PEEP value, the difference between ${C}_{\mathrm{max}}$ and its compliance is computed as $\Delta C$, which quantifies the degree of atelectasis or overdistension. The tissue classification image is derived by applying this analysis to all pixels and can guide the selection of an optimal PEEP value to recruit collapsed lungs with a minimal amount of overdistension [17, 41, 42].

### Parameters of spatial air distribution

The anterior-to-posterior ventilation ratio ($\mathrm{A/P}$ ratio) is a measure of general distribution characteristics and calculated using the sums of the pixel values in the ventral and dorsal regions [43]. The $\mathrm{A/P}$ ratio expresses any changes in the regional ventilation along the vertical direction.

The center of ventilation ($\mathrm{CoV}$) is defined following the concept of the center of gravity in mechanics. The ${\mathrm{CoV}}_{{x}}$ and ${\mathrm{CoV}}_{{y}}$ in (3) are the ratios of the *x*- and *y*-weighted pixel sums to the global pixel sum, respectively, where *x* and *y* are coordinates of the pixel:

3 $${\text{CoV}}_{x} \,[\% ]\, = \,\frac{{\sum {(x_{j} \, \times \,V_{j} )} }}{{\sum {V_{j} } }}\quad{\text{ and \quad CoV}}_{y} \,[\% ]\, = \,\frac{{\sum {(y_{j} \, \times \,V_{j} )} }}{{\sum {V_{j} } }}.$$

where $x_{j}$ and $y_{j}$ are the horizontal position (*x*-axis) and vertical position (*y*-axis) of pixel *j*. The ${\mathrm{CoV}}_{{x}}$ and ${\mathrm{CoV}}_{{y}}$ increase from 0% at the top-left corner and approach 100% at the bottom-right corner [22].

The global inhomogeneity index ($\mathrm{GI}$) in (4) expresses the degree of inhomogeneous air distributions in the lungs [44]. The $\mathrm{GI}$ value is computed by normalizing the total deviation from the median value by the sum of pixel values:

4 $${\text{GI}}\, = \,\frac{{\sum {\left| {V_{j} - {\text{Median}}\,(V)} \right|} }}{{\sum {V_{j} } }}.$$

The coefficient of variation ($\mathrm{CV}$) is a statistical measure defined as the ratio of the standard deviation over the mean. The $\mathrm{CV}$ value is used to express the heterogeneity of the air distributions in the lungs for obstructive pulmonary diseases [45].

### Animal experiments

**Fig. 11:**
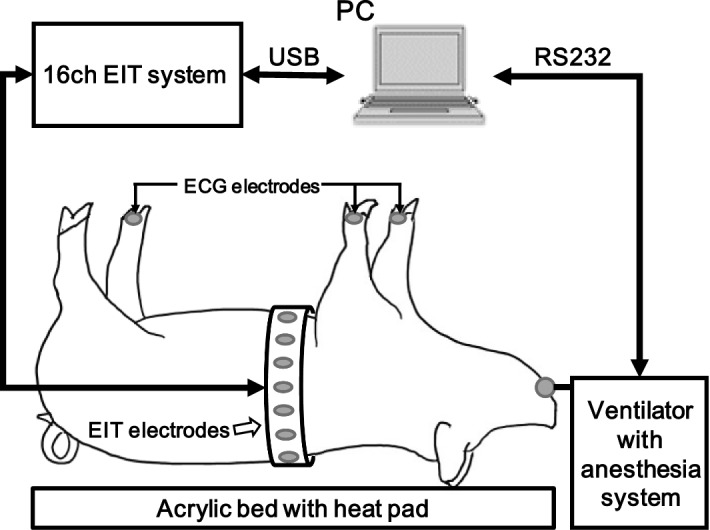
Experimental setup

The animal experiments using ten normal pigs (age: 6 months, weight: 29.5 ± 4.4 kg, chest circumferences: 64 ± 9 cm) were conducted in accordance with all regulations of the Institutional Animal Care and Use Committee (KBIO IACUC 2017035). The animal was intravenously anesthetized using a syringe pump (0.5 mL/kg IV injection of 4:1 ketamine and xylazine mixture) and connected to a mechanical ventilator (Hamilton-G5, Hamilton Medical, Switzerland) by tracheal intubation. The status of the animal was continuously monitored using a patient monitor (IntelliVue MP50, Philips, The Netherlands). The chest hair was removed and 16 Ag/AgCl electrodes were attached at the fifth intercostal space for EIT imaging (Fig. 11). To obtain EIT images with proper anatomical structural information, the chest shape and electrode positions were measured using a 3D scanner (Sense, 3D Systems, U.S.). EIT data were collected by injecting 1 ${\mathrm{mA}}_{\mathrm{pp}}$ currents at 11.25 kHz using a 16-channel KHU Mark 2.5 EIT device with a temporal resolution of 50 frames/s [31]. Adopting the adjacent current-injection and voltage-measurement protocol, each EIT image was reconstructed using 208 boundary voltage data excluding the voltages measured between any pairs of electrodes used for current injections. The collected EIT data were synchronized with the airway volume and pressure data from the mechanical ventilator. For EIT image reconstructions, we used the fidelity-embedded regularization (FER) method with a subspace-based motion artifact removal algorithm [46].

To validate the operation of integrated EIT system for monitoring of lung functions using 11 functional images and measures, the estimated air volume from the time-series of TV images was evaluated with the tidal volume controlled in mechanical ventilator at the first experiment. The TV images were produced from the EIT image while increasing the tidal volume from 100 to 600 mL. We performed a linear analysis and a Bland–Altman analysis using the pixel sum in the TV images and the measured global TV data from the ventilator.

Three functional images of TV, $\triangle$EELV, and compliance in the real-time screen view were validated when applying the PEEP titration protocol for normal pigs. Refer to the $0~{\mathrm{cmH}}_2 \mathrm{O}$, we increased PEEP level from 5 to $25~{\mathrm{cmH}}_2 \mathrm{O}$ with $5~ {\mathrm{cmH}}_2\mathrm{O}$ step. After that, we apply a decremental PEEP in the opposite direction when recording the EIT and ventilator data in the integrated EIT system continuously. Here, we excluded the ventilation delay image because we could not watch any change in the normal pigs. From the ventilator, we could get the global compliance value when dividing the pressure data by volume data. We could compare it to the sum of pixels in the compliance image for validating the process of compliance images.

We used the lung-collapsed animal model to verify TV, $\triangle$EELV, ventilation delay image, and air distribution parameters ($\mathrm{A/P}$ ratio, ${\mathrm{CoV}}_{{x}}$, ${\mathrm{CoV}}_{{y}}$, GI, CV). Here, we used ventilation image which added TV and $\triangle$EELV images. For five pigs, regional lung collapse was induced using the saline lavage method to wash out surfactant from parts of the lungs. The presence of regional lung collapse was examined by X-ray CT images and the arterial blood gas analysis. Switched to the pressure control mode, the PEEP titration was applied to find the optimal PEEP level for each case. We watched the region changes for overdistended and collapsed lung when applying the PEEP titration in the analysis screen view. Optimal PEEP was chosen at the PEEP level when it could minimize both overdistension and collapse values.

## Data Availability

The datasets used and/or analyzed during the current study are available from the corresponding author on reasonable request.

## Abbreviations

A/P ratio: anterior-to-posterior ventilation ratio
CoV: center of ventilation
Crs: respiratory system compliance
CV: coefficient of variation
EELV: end-expiratory lung volume
EIT: electrical impedance tomography
FER: fidelity-embedded regularization
GI: inhomogeneity index
ICU: intensive care unit
LPV: lung protective ventilation
PEEP: positive end-expiratory pressure
RR: respiration rate
TV: tidal volume
